# AQEA-QAS: An Adaptive Quantum Evolutionary Algorithm for Quantum Architecture Search

**DOI:** 10.3390/e27070733

**Published:** 2025-07-08

**Authors:** Yaochong Li, Jing Zhang, Rigui Zhou, Yi Qu, Ruiqing Xu

**Affiliations:** 1 College of Information Engineering, Shanghai Maritime University, 1550 Haigang Avenue, Pudong New Area, Shanghai 201306, China; ycli@shmtu.edu.cn (Y.L.); rgzhou@shmtu.edu.cn (R.Z.); quyiqq@aliyun.com (Y.Q.); 2 Research Center of Intelligent Information Processing and Quantum Intelligent Computing, Shanghai Maritime University, 1550 Haigang Avenue, Pudong New Area, Shanghai 201306, China; 3 Faculty of Intelligence Technology, Shanghai Institute of Technology, 100 Haiquan Road, Fengxian District, Shanghai 201418, China; xrq@sit.edu.cn

**Keywords:** quantum computing, quantum neural network, quantum evolutionary algorithm

## Abstract

Quantum neural networks (QNNs) represent an emerging technology that uses a quantum computer for neural network computations. The QNNs have demonstrated potential advantages over classical neural networks in certain tasks. As a core component of a QNN, the parameterized quantum circuit (PQC) plays a crucial role in determining the QNN’s overall performance. However, quantum circuit architectures designed manually based on experience or using specific hardware structures can suffer from inefficiency due to the introduction of redundant quantum gates, which amplifies the impact of noise on system performance. Recent studies have suggested that the advantages of quantum evolutionary algorithms (QEAs) in terms of precision and convergence speed can provide an effective solution to quantum circuit architecture-related problems. Currently, most QEAs adopt a fixed rotation mode in the evolution process, and a lack of an adaptive updating mode can cause the QEAs to fall into a local optimum and make it difficult for them to converge. To address these problems, this study proposes an adaptive quantum evolution algorithm (AQEA). First, an adaptive mechanism is introduced to the evolution process, and the strategy of combining two dynamic rotation angles is adopted. Second, to prevent the fluctuations of the population’s offspring, the elite retention of the parents is used to ensure the inheritance of good genes. Finally, when the population falls into a local optimum, a quantum catastrophe mechanism is employed to break the current population state. The experimental results show that compared with the QNN structure based on manual design and QEA search, the proposed AQEA can reduce the number of network parameters by up to 20% and increase the accuracy by 7.21%. Moreover, in noisy environments, the AQEA-optimized circuit outperforms traditional circuits in maintaining high fidelity, and its excellent noise resistance provides strong support for the reliability of quantum computing.

## 1. Introduction

Over recent decades, quantum neural networks (QNNs) have emerged as a research hotspot at the intersection of the fields of quantum computing and artificial intelligence, experiencing significant development. The concept of QNNs was first proposed in the 1990s [1], and its application potential was demonstrated theoretically. For instance, Hogg and Shor et al. [2,3] laid the foundation for QNN development by presenting the basic framework of quantum search and optimization algorithms. With the advancement of quantum computing technology, researchers have gradually integrated the QNN models with fault-tolerant quantum computing, aiming to achieve stable quantum learning systems in noisy environments [4]. However, with the recent advent of near-term intermediate-scale quantum (NISQ) devices, QNN research has entered a new phase. Although the NISQ devices cannot perform fully fault-tolerant quantum computing, they can provide sufficient computational power to support many practical applications. Against the backdrop of the NISQ, a series of representative works on the QNN design have been conducted, and the advantages of the QNN in certain specific tasks have been demonstrated. For instance, the quantum approximate optimization algorithm (QAOA) proposed by Farhi et al. [5] performs well in solving combinatorial optimization problems. Building on this, Wiersema et al. [6] optimized QAOA variational ansätz to address optimization plateaus via overparameterization, Matos et al. [7] quantified QAOA efficiency using interaction distance metrics for state preparation, and Sun et al. [8] enhanced QAOA for non-symmetric states with a parameterized Hamiltonian framework. These studies have significantly advanced QAOA’s capabilities, offering valuable insights for its applications in quantum computing and physics. In addition, Havlicek et al. [9] demonstrated that a quantum-enhanced support vector machine could outperform classical algorithms in image classification tasks. In the QNN design, the construction of a parameterized quantum circuit (PQC) represents the main challenge [10]. A well-designed PQC can not only effectively represent complex quantum states but also increase training efficiency and improve the generalization ability of a model during the optimization process [11]. However, this issue also faces numerous challenges, such as selecting an appropriate circuit depth and suitable parameterization strategies, all of which have a significant influence on a QNN’s performance. A well-designed circuit architecture can avoid barren plateaus and accelerate the training process of the PQC [12,13,14,15]. In view of that, recent studies have proposed quantum architecture search (QAS) algorithms as a solution to the automatic design of high-performance quantum circuit structures [16,17].

The QAS algorithms can automatically design quantum circuits for a given task, and their main aim is to find high-performance quantum circuits for variational quantum algorithms using classical optimization approaches. The QAS algorithms mainly consist of two parts: a search module and an evaluation module. The search module uses various search strategies to explore the optimal quantum circuits in the search space, whereas the evaluation module measures the real performance of different quantum circuits and guides the search module to explore high-performance circuits in the search space. The QAS algorithms can be roughly classified into algorithms with and without machine learning-based models according to the adopted search kernel algorithms. The algorithms without machine learning-based models include the RotoSelect algorithm [18], the Variable Ansatz (VAns) algorithm [19], the Quanto algorithm [20], and other algorithms [21,22]. In addition, these algorithms include the search algorithms that employ intelligent algorithms, such as greedy algorithms [23] and simulated annealing algorithms [24], as a core. In algorithms that use machine learning-based models, the relationship between the structure selection process and the structure’s performance in a specific task is an unknown black-box relationship because the performance of a quantum circuit structure is highly related to the particular task. Currently, most studies on the QAS algorithms focus on the search module, and different search strategies have been proposed, including gradient-based algorithms, reinforcement learning [25,26,27,28], and evolutionary algorithms [29,30,31].

In quantum evolutionary algorithms, the update process of a quantum population represents the main step. The quantum population updating mostly relies on a fixed rotation angle table to fix the rotation angle’s value, and the algorithm needs to compare the current state with the optimal state during the evolutionary process. However, after many evolutionary iterations, differences between the individual chromosomes in a quantum population can become increasingly large. Considering this situation, this study proposes an adaptive quantum evolutionary algorithm (AQEA) to improve the algorithm’s performance. Instead of using fixed rotation angles, this study employs two dynamic rotation angle strategies to overcome the problem of larger differences between individuals when the number of evolutions increases. In addition, an elite parent retention strategy, where the parent and offspring are considered together as a new parent when selecting the new offspring, is employed. Furthermore, the quantum catastrophe strategy, which can assist the population in breaking the current stagnant state when the algorithm falls into a local optimum, is introduced.

The main contributions of this work can be summarized as follows:

1. The QNN circuit structure for the QEA search has certain redundancy. Aiming to address this problem, this study introduces two innovative optimization strategies, which can effectively reduce the complexity of the generated circuits and minimize the usage of unnecessary gates, achieving a 17.39% reduction in the number of quantum gates;
2. Due to the inherent randomness and limited search iterations, the QEAs are prone to fall into a local optimum. To overcome these challenges, this study introduces two strategies: an elite parent retention strategy and a quantum catastrophe strategy. The former ensures the preservation of superior genetic traits in a population, whereas the latter facilitates the algorithm’s escaping process from the local optima it fell into;
3. The QNN structure search by the proposed AQEA is experimentally conducted on the MNIST, Fashion-MNIST, and CIFAR-10 datasets. Compared with the QNN based on the QEA and the original QNN, the proposed AQEA can improve the accuracy by 7.21%, 3.95%, and 2.35%, respectively;
4. The circuit architectures optimized by the proposed AQEA for different datasets consistently achieve significantly higher fidelity than conventional template circuits under various noise types and noise probabilities, showcasing stronger noise adaptability and robustness, thus providing an optimal solution for quantum circuit design in noisy scenarios.

The remainder of this paper is structured as follows. Section 2 introduces the basic concepts of quantum computing and presents a detailed exposition of the related work in QAS. Section 3 elaborates on QNNs and each step of the AQEA searching the QNN structure, from the encoding phase to the end step of the algorithm. Section 4 verifies the effectiveness and noise resistance of the proposed AQEA. Section 5 concludes this study and discusses future work.

## 2. Preliminaries

### 2.1. Quantum Computing

Basic knowledge related to quantum computing can be found in [32]. Here, only the quantum gates that are mainly used in this paper are introduced, such as XX(θ), YY(θ), ZZ(θ) gates, expressed by:

(1) $$XX\left(\theta \right)=\left[\begin{array}{cccc} \cos(\frac{\theta }{2}) & 0 & 0 & -i\sin(\frac{\theta }{2})\\ 0 & \cos(\frac{\theta }{2}) & -i\sin(\frac{\theta }{2}) & 0\\ 0 & -i\sin(\frac{\theta }{2}) & \cos(\frac{\theta }{2}) & 0\\ -i\sin(\frac{\theta }{2}) & 0 & 0 & \cos(\frac{\theta }{2}) \end{array}\right],$$

(2) $$YY\left(\theta \right)=\left[\begin{array}{cccc} \cos(\frac{\theta }{2}) & 0 & 0 & i\sin(\frac{\theta }{2})\\ 0 & \cos(\frac{\theta }{2}) & -i\sin(\frac{\theta }{2}) & 0\\ 0 & -i\sin(\frac{\theta }{2}) & \cos(\frac{\theta }{2}) & 0\\ i\sin(\frac{\theta }{2}) & 0 & 0 & \cos(\frac{\theta }{2}) \end{array}\right],$$

(3) $$ZZ\left(\theta \right)=\left[\begin{array}{cccc} e^{-\frac{i\theta }{2}} & 0 & 0 & 0\\ 0 & e^{\frac{i\theta }{2}} & 0 & 0\\ 0 & 0 & e^{\frac{i\theta }{2}} & 0\\ 0 & 0 & 0 & e^{-\frac{i\theta }{2}} \end{array}\right].$$

The parameterized quantum logic gates act on qubits to change their mapped positions on the Bloch sphere. Namely, they deflect a qubit along the *X*, *Y*, and *Z* axes to alter its quantum state, with rotation angles θ, defining the gate parameters that enable precise manipulation of superposition and entanglement in quantum algorithms.

In the field of quantum machine learning, angle encoding is often employed to map input data onto quantum states, which allows for efficient pattern recognition and classification using quantum algorithms. For instance, in [33], the authors proposed a feature mapping method using quantum angle encoding, which could effectively enhance the performance of quantum support vector machine (QSVM), improving the classification accuracy compared with traditional support vector machine (SVM). In addition, angle encoding was employed to solve combinatorial optimization problems. For an *N*-dimensional classical vector $x=\left[\begin{array}{c} x_{1},x_{2},\ldots ,x_{N} \end{array}\right]$, each component $x_{i}$ can be mapped to the rotation angle of a qubit. Particularly, a rotation gate $R_{y}$ can be used to encode data into quantum states as follows:

(4) $$R_{y}\left(x_{i}\right)=\left[\begin{array}{cc} \cos\left(\frac{x_{i}}{2}\right) & -\sin\left(\frac{x_{i}}{2}\right)\\ \sin\left(\frac{x_{i}}{2}\right) & \cos\left(\frac{x_{i}}{2}\right) \end{array}\right]\left[\begin{array}{c} 1\\ 0 \end{array}\right]=\cos\left(\frac{x_{i}}{2}\right)|0\rangle +\sin\left(\frac{x_{i}}{2}\right)|1\rangle ,$$

### 2.2. Related Work

Most existing QAS algorithms estimate a quantum circuit’s performance by optimizing the gate parameters using the gradient descent method, which can be highly time-consuming because the gate parameters need to be updated numerous times until the algorithm converges. Wang et al. [34] proposed a super circuit with shared gates to accelerate the circuit evaluation process and estimated the performance of quantum circuits directly using the gate parameters from a super circuit trained with all candidate circuit architectures. Another method for accelerating circuit evaluation based on a performance predictor trained on a small number of circuit architectures and their corresponding true performance was proposed in [35]. The numerical simulation results showed that the prediction performance of this method had a high correlation with the true performance, and it could find excellent variational quantum solver architectures. The quantum circuit architectures have also been proven to perform well in image classification tasks. In addition, He et al. [36] used information on the gradients of parameterized circuits to reduce the computational burden associated with evaluating candidate architectures in QASs, thus enabling more efficient exploration of the design space in quantum computing tasks. A self-supervised Bayesian QAS framework [37] was later proposed, integrating graph learning to enhance prediction efficiency via structured latent space modeling. Furthermore, to accelerate the QAS algorithms, Zhang et al. [38] proposed a probabilistic model to relax the discrete search space of quantum circuits to a continuous domain and optimize their architectures using gradient descent. Additionally, formal methods like integrating ZX-calculus with genetic programming are used to optimize parameterized quantum circuits for more efficient QAS [39]. Xie et al. [40] proposed DeQompile, a genetic programming framework that decompiles low-level quantum circuits into interpretable Qiskit algorithms via abstract syntax trees, addressing the explainability bottleneck in QAS. This approach validates its efficacy in reconstructing circuit patterns and balancing explainability-efficiency trade-offs across benchmark quantum algorithms. A recent training-free QAS framework utilizes landscape fluctuation analysis to efficiently evaluate circuits without costly training [41].

There are also QASs based on gradient-free search strategies, such as reinforcement learning-based and evolutionary algorithms, which can find high-performance quantum circuits. In [42], the authors designed a deep reinforcement learning (DRL)-based model for quantum circuit optimization, which includes an agent whose task is to interact with the environment to optimize a particular quantum circuit. Further studies have utilized reinforcement learning to optimize quantum circuits, addressing the noise and limitations of NISQ-era hardware and demonstrating effectiveness in noisy environments [43,44,45]. He et al. [46] developed a MetaQAS algorithm that uses the existing experience to search for circuit architectures. The MetaQAS learns architecture and gate parameter initialization heuristics from many training tasks and embeds cross-knowledge. Furthermore, using the initialization heuristic algorithm, the QAS algorithm can converge after a few updates in new tasks. Nevertheless, there are adaptive designs of quantum circuit structures that employ classical optimization methods [47]. When facing new tasks, the search algorithm cannot effectively use the prior experience from previous tasks, which results in low search efficiency. To improve the search efficiency of QAS algorithms, recent research has proposed using meta-learning-based methods [48]. Beyond traditional search strategies, generative models like denoising diffusion models are emerging for quantum circuit synthesis, efficiently producing circuits by overcoming classical simulation overheads [49].

Recently, inspired by evolutionary algorithms, some studies have encoded a quantum circuit as a genetic sequence and employed quantum evolutionary algorithms for optimization. In this approach, through continuous evolution in each generation, an excellent individual is selected, and the process continues until the optimal quantum circuit structure is found. Li et al. [50] proposed an evolutionary quantum neural architecture search (EQNAS) method, which optimizes the design of a QNN using a quantum evolutionary algorithm. This method encodes input images through quantum encoding and initializes a quantum population, and the fitness evaluation and population updates are used to find the best network structure. The experimental results have shown that the EQNAS method can effectively reduce the complexity of a quantum circuit and improve classification accuracy. In 2024, Li et al. [51] developed a quantum evolutionary algorithm for the structure design of a quantum convolutional neural network (QCNN). They regarded the QCNN design process as a combinatorial optimization problem and used the global search capability of the quantum evolutionary algorithm to design structures adaptively in a large-scale discrete search space. The experiments have demonstrated that this method can simplify the QCNN circuit design while enhancing its expressive power.

## 3. Proposed Method

### 3.1. QNN

A quantum circuit represents a fundamental framework and a basic structure for constructing quantum algorithms. The QNNs consist of quantum circuits and various quantum logic gates built upon them. Since the quantum circuits in QNNs contain adjustable parameters, they are commonly referred to as parameterized quantum circuits. Similar to classical circuits, quantum circuits intuitively represent critical information, such as the structure type and complexity level of quantum algorithms.

**Figure 1:**
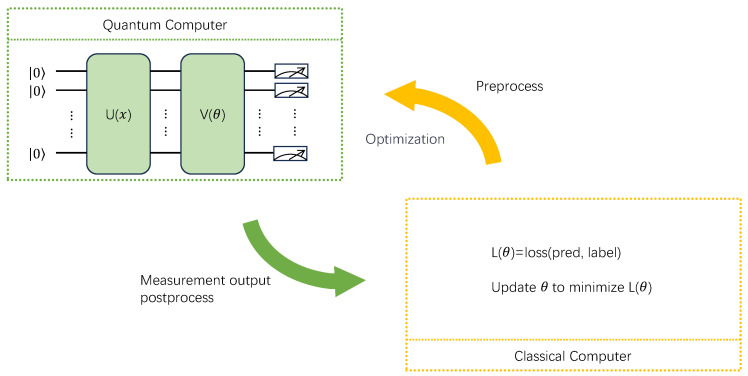
Illustration of a hybrid classical-quantum computing architecture. Classical computers handle the optimization of features and loss functions before inputting data into QNNs, while quantum computers focus on training and optimizing parameters within the network architecture, with both systems collaborating to complete the task.

The PQC denotes a quantum computing model that uses adjustable parameters to construct quantum states, which can be optimized during the learning process. The PQC often needs to collaborate with classical networks or optimizers running on a computer to adjust a model’s parameters and train the model. The parameters optimization process of the PQC employs classical algorithms to evaluate a model’s performance and guide the model parameters’ update process. Therefore, in practical applications, the PQC is typically used as part of a quantum-classical hybrid algorithm. The application of the PQC enables quantum hardware to perform quantum state preparation and evolution while using regular computers for data processing and optimization, thus allowing the execution of quantum machine learning tasks. The process where a quantum computer and a classical computer collaborate to complete a learning task is illustrated in Figure 1.

**Figure 2:**
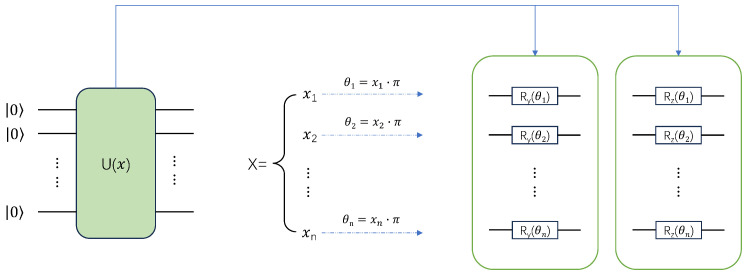
The data encoding layer of the QNN model. This is an essential component in the QNN model, responsible for converting classical data into quantum states that can be processed on quantum computers, enabling classical information to be efficiently loaded into quantum systems for further quantum computational operations.

As shown in Figure 1, before data are sent to the quantum circuit, due to the limited quantum computing resources, the data dimension needs to be reduced. This process is called data preprocessing. During the data preprocessing process, classical network modules can be used for feature extraction, such as convolutional neural networks, multi-layer perceptrons, or various machine learning-based algorithms, including principal component analysis and support vector machines. After dimensionality reduction, the data are fed to a QNN consisting of three modules; the first encoding module is depicted in Figure 2.

In the first encoding module, the preprocessed data are encoded into a quantum state using U(x). In Figure 2, on the left side of U(x) is the input quantum state. In this study, 10 qubits are used, with the initial state of all 10 qubits set to the $|0\rangle$ state by default; U(x) contains two layers of rotation gates, a layer of $R_{y}$ gates, and a layer of $R_{z}$ gates. It should be noted that using two layers of rotation gates can enhance the expressive power of a quantum circuit. Namely, through multiple rotations, the quantum state can be adjusted more precisely, allowing for better capturing of the complex structure of data. After the mapping process, the input vector is converted into rotation angles to guide the rotation process of the qubits, which are then sent to the second module parameter quantum circuit of the QNN.

**Figure 3:**
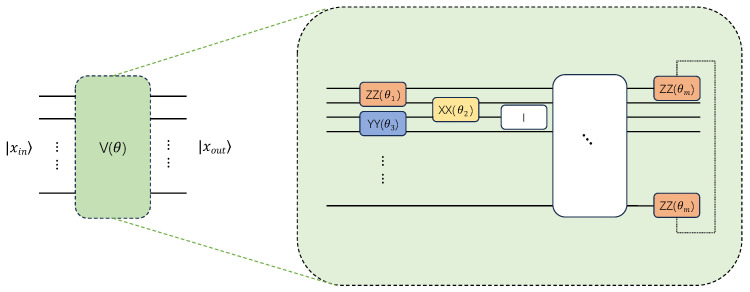
The variational layer of the QNN model. This component is critical in the QNN model, whose architecture is typically manually designed. In this study, the circuit architecture of the variational layer is obtained through the AQEA search method proposed in the next section.

The most important part of the QNN model, the variational layer, is displayed in Figure 3. In this layer, the placement and types of all quantum logic gates are determined by the AQEA, which is explained in detail in Section 3.2. However, it should be noted that the parameter gates in this layer differ from those in the encoding layer. Namely, in the encoding layer, the parameters are usually fixed and stay fixed after being defined unless the input data changes. By contrast, the parameters of the variational layer are updated, typically using the gradient descent method or other optimization algorithms, with the aim of minimizing the loss function value. During training, the parameters are gradually optimized based on feedback until the algorithm convergences to an ideal state.

Next, the measurement of the quantum system is performed, causing the data to collapse into a classical state. The measurement operation is realized using the Pauli *Z* basis. The circuit for measuring with the Pauli *Z* basis is used to obtain the expected value of the quantum state relative to the Pauli *Z* operator. Specifically, the two eigenstates of the Pauli *Z* operator are $|0\rangle$ and $|1\rangle$, and its effect is $Z|0\rangle$ = $|0\rangle$, $Z|1\rangle$ = $-|1\rangle$, with corresponding eigenvalues of +1 and −1. For an arbitrary mixed state of a qubit $|\psi \rangle$, $|\psi \rangle$ = $\alpha |0\rangle$ + $\beta |1\rangle$, and it satisfies ${\left|\alpha \right|}^{2}+{\left|\beta \right|}^{2}=1$. Then the expected value of this qubit $|\psi \rangle$ under the Pauli *Z* operator is

(5) $$\begin{array}{cc} \langle Z\rangle & =\langle \psi |Z|\psi \rangle =(\alpha \langle 0|+\beta \langle 1|)Z(\alpha |0\rangle +\beta |1\rangle )=(\alpha \langle 0|+\beta \langle 1|)(\alpha |0\rangle -\beta |1\rangle )\\ \; & ={\left|\alpha \right|}^{2}-{\left|\beta \right|}^{2} \end{array}.$$

### 3.2. AQEA for QNNs

#### 3.2.1. AQEA Overview

**Figure 4:**
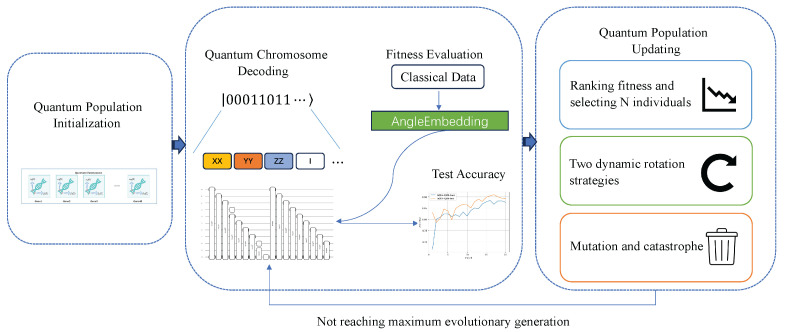
The sequence of steps of the proposed AQEA. The process begins with quantum population initialization, followed by observation of the quantum population to collapse into definitive quantum individuals after initialization. These individuals are then decoded into corresponding quantum circuits, with each circuit evaluated to obtain fitness values. Subsequently, the quantum population is iteratively updated based on the fitness values until the predefined maximum number of evolutionary iterations is reached.

The proposed AQEA can be roughly divided into several parts as follows. First, the quantum population initialization is performed, and related parameters that affect the population change, such as the population size, gene length, maximum number of evolutions, the coefficient related to the rotation angle, and the catastrophe factor, are set. Next, the observation of the initialized quantum population is conducted, which simulates the measurement operation in a quantum network. The purpose of this process is to obtain a definite quantum population state, which defines the search space of a quantum logic gate. Next, quantum circuits are created in sequence based on the obtained quantum population. In this study, the term quantum circuit refers to the above-mentioned variational layer, and the arrangement order of quantum gates in the encoding layer is prepared in advance and does not change during the algorithm updating process. The fourth step is the evaluation of the population fitness value, where the fitness selection is based on the accuracy rate of the quantum circuits created by the population individuals on the test dataset. The quality of the population of individuals is judged based on the accuracy rate. The last and most important step is the quantum population update. In this study, an adaptive mechanism is proposed to update the quantum population, and two dynamic rotation angle update strategies, as well as a mutation strategy, are designed using the number of evolutions and the fitness value of the individuals to update the quantum population. Meanwhile, during the evolution process, to prevent the population from developing in a poor direction, the parent generation and the evaluated offspring generation are combined at each iteration to select the next generation. In addition, a catastrophe factor is introduced to monitor whether the population has fallen into a local optimum and to activate the quantum catastrophe strategy to break the current state when needed, thus continuing to search for the optimal architecture. The flowchart of the proposed algorithm is presented in Figure 4.

#### 3.2.2. Quantum Chromosome Encoding

In traditional evolutionary algorithms (e.g., genetic algorithms), chromosome encoding typically adopts binary encoding, real number encoding, or symbolic encoding, which directly maps input data to the variable values in the solution space of the problem. For instance, binary encoding represents a chromosome as a string composed of zeros and ones and defines the characteristics of an individual through the position and value of the genes. Although this encoding method is simple and easy to implement, it has a few limitations. First, the expressive power of traditional encoding is restricted by the encoding length, where longer encodings increase the computational complexity, whereas shorter encodings can yield insufficient solution accuracy. Second, a fixed representation of chromosomes makes them prone to fall into a local optimum when handling complex problems, and due to the limited expressive power, it is challenging to explore the solution space of multimodal problems efficiently. By contrast, the quantum chromosome encoding method uses the superposition state of qubits and quantum gate operations to represent the solution space. Quantum chromosomes describe an individual’s state using the probability amplitudes of qubits and can simultaneously represent multiple possible solutions. This ability to use parallel representation significantly enhances the search scope. In this study, a quantum chromosome is encoded using 40 qubits, and the qubits on a chromosome can be referred to as genes. Each qubit on a quantum chromosome is not fixed at zero or one but is in an uncertain state. A quantum chromosome can be defined as follows:

(6) $$q=\left[\begin{array}{cccc} \alpha_{1} & \alpha_{2} & \ldots & \alpha_{n}\\ \beta_{1} & \beta_{2} & \ldots & \beta_{n} \end{array}\right],$$

where *n* represents both the length of a quantum chromosome and the number of qubits; ${\left|\alpha \right|}^{2}+{\left|\beta \right|}^{2}=1$, where $\alpha_{i}$ and $\beta_{i}$ represent the probability amplitudes of the *i*th qubit being zero and one, respectively.

Therefore, a quantum chromosome can also be expressed by

(7) $$q=\left[\begin{array}{cccc} \cos(\theta_{1}) & \cos(\theta_{2}) & \ldots & \cos(\theta_{n})\\ \sin(\theta_{1}) & \sin(\theta_{2}) & \ldots & \sin(\theta_{n}) \end{array}\right].$$

Since a qubit has a state of both zero and one simultaneously before it collapses into a definite state upon observation, it is necessary to analyze the quantum population to obtain definite quantum individuals. The quantum population initialization and observation algorithm is presented in Algorithm 1.

In this study, it is assumed that each individual in the quantum population has 40 genes, which is equivalent to 40 qubits, so there are a total of $2^{40}$ possibilities. Furthermore, since two qubits can be regarded as a whole, 40 qubits denote 20 qubit pairs. Considering that two qubits can represent the four states of $|00\rangle$, $|01\rangle$, $|10\rangle$, and $|11\rangle$, for the above-mentioned combinations, four different decoding methods are designed in the quantum circuit, as shown in Table 1.
**Algorithm 1** Quantum population initialization and observation.**Input:** The size of quantum population *N*, the length of a quantum chromosome *L*, empty quantum population;**Output:** observed quantum population $Q_{out}$; 1:**for** *q* in $Q_{in}$ **do** 2:      i=1 3:      r=0.5 4:      **while** j<=L **do** 5:            θ=random(0,90) 6:            $\alpha_{j}=\cos\theta$ 7:            $\beta_{j}=\sin\theta$ 8:            **if** $r<\alpha_{j}^{2}$ **then** 9:                 $q_{j}=0$10:            **else**11:                 $q_{j}=1$12:            **end if**13:            i=i+114:      **end while**15:**end for**16:**return** $Q_{out}$

The final quantum state of each qubit pair denotes the selected quantum gate. For instance, if the first and second gene positions are observed to be both zeros, then an XX gate will be placed at the first qubit and the auxiliary qubit position when creating a quantum circuit; if the two positions are zero and one, then a YY gate will be placed, and so on. The second and third gene positions correspond to placing an appropriate gate at the second qubit and the auxiliary qubit position in the circuit. This process continues until all the gene positions of a quantum chromosome are defined, which indicates that the quantum circuit architecture is decided. It can be seen that there are at most 20 quantum gates in the quantum circuit, which might also include non-parameter and identity gates. In this study, the quantum gates in the encoding layer are neglected, and only the number of trainable gates is considered.

The fitness value represents an indicator that measures an individual’s ability to adapt to a specific environment, and the fitness evaluation process is a standard method for assessing an individual’s overall quality. The level of an individual’s fitness directly defines their superiority or inferiority. However, to determine the superiority or inferiority of an individual, two key calculation steps should be conducted: (1) calculate the objective function value, which denotes the result of the individual after the encoding process; (2) construct a suitable fitness function based on the objective function values according to the specific rules to determine the fitness score of the individual. The fitness value not only reflects an individual’s performance in a given task but also plays a role in the screening and optimization tasks throughout the evolutionary process. In this study, the image classification task is considered, so the accuracy of a model on the test dataset is used as a direct indicator in the fitness evaluation of individuals. In each iteration of the evolutionary algorithm, each individual (i.e., a circuit) is trained and applied to the test dataset. The performance of each individual on the test dataset is recorded as a fitness score. Based on the obtained fitness score values, a selection operation is performed, prioritizing the retention of individuals with high accuracy while eliminating individuals with poor performance. This process guides the population toward a better solution. As the evolutionary algorithm iterates, individuals with low fitness values are eliminated, so the overall fitness score of the population gradually improves with time. The pseudo-code of the fitness evaluation algorithm for a quantum population is presented in Algorithm 2.
**Algorithm 2** Fitness evaluation of quantum population.**Input:** A quantum population by observed *Q*, the number of training epochs *N*;**Output:** A fitness value $f_{i}$ of a quantum population 1:**for** *q* in *Q* **do** 2:      i=1 3:      Embed classical data to the quantum state by the encoding layer 4:      Decode *q* to a circuit architecture with parameters defined by $\theta =\{XX\left(\theta_{1}\right),YY\left(\theta_{2}\right),ZZ\left(\theta_{3}\right),\ldots \}$ 5:      **while** i<=N **do** 6:            Initialize parameters of quantum gates θ in the quantum circuit 7:            Train the parameterized quantum circuit using the Adam optimizer 8:            Calculate the loss and updating parameters $\theta \to \theta^{\prime }$ 9:            Evaluate the model performance and calculate accuracy Acc10:            i=i+111:      **end while**12:      Preserve maximum Acc and convert to fitness $f_{i}$13:**end for**14:**return** $f_{i}$

In Algorithm 2, before the loss value is calculated, the expected value obtained by the measurement process of the Pauli *Z* basis described before is not directly used in the loss calculation process. The expected value obtained by the measurement ranges from −1 to 1, whereas the label values of the dataset are all positive, but they are converted to zeros and ones before being input into the quantum circuit. Therefore, the expected value of the measurement needs to be transformed into a probability distribution using the sigmoid function. In this study, the binary cross-entropy loss function is selected; this function measures the difference between the predicted probability distribution of a model and the true labels, and it is suitable for binary classification problems where the output is either zero or one. The binary cross-entropy loss function is expressed as follows:

(8) $$L\left(y,\hat{y}\right)=-\left[y\log\left(\hat{y}\right)+\left(1-y\right)\log\left(1-\hat{y}\right)\right],$$

where *y* represents the true label of data (0 or 1), and $\hat{y}$ is the predicted probability of the model for these data; when y=1, the loss is mainly determined by the first term in the above-mentioned equation, that is, $-\log(\hat{y})$, and the loss gradually decreases; otherwise, the loss gradually increases.

#### 3.2.3. Quantum Population Updating

In evolutionary algorithms, population update is a fundamental step to complete the algorithm iteration and optimization process. The population update process mainly includes three basic operations: selection, crossover, and mutation. First, the selection operation screens individuals based on their fitness values, where individuals with higher fitness have a higher possibility of being selected for the next generation. Common selection methods include the roulette wheel selection method and the tournament selection method, which ensure that genetic information on superior individuals is retained and passed on using different strategies. Then, the crossover operation exchanges gene segments between the selected individuals. Through the crossover operation, the superior genes of individuals are combined to generate new individuals, which helps to explore the solution space and increase the population’s diversity. The implementation methods of crossover are diverse, including the single-point crossover method, the multi-point crossover method, and the uniform crossover method. Finally, the mutation operation randomly alters the genes of the individuals. The mutation operation is a random perturbation in the population’s update, which can break the local optimal state of the population and, thus, prevent the algorithm from converging prematurely.

During the update process of the quantum evolutionary algorithm, the population adjustment is mainly achieved through the unique quantum rotation gate adjustment using quantum theory. The proposed AQEA employs two adaptive quantum rotation gate angle strategies for rotation update and introduces a quantum *X* gate to achieve the quantum population’s mutation; in addition, the quantum catastrophe idea is adopted to assist the algorithm in escaping from a local optimum with a certain probability after getting trapped in it [52,53] and to continue to search the global solution.

In the quantum evolutionary algorithm, quantum transformation matrices are used to achieve changes between the quantum superposition states and entangled states. The generation result of offspring individuals is not defined by the genes of the parent generation but by the probability amplitude of their states. The quantum rotation gate is mainly used to perform the generation process, and the rotation angle of the quantum rotation gate is used to calculate the probability of the chromosome mutation operation as follows:

(9) $$U\left(\Delta \theta \right)=\left[\begin{array}{cc} \cos(\Delta \theta ) & -\sin(\Delta \theta )\\ \sin(\Delta \theta ) & \cos(\Delta \theta ) \end{array}\right],$$

where Δθ is the rotation angle and can typically be determined from Table 2.

In Table 2, $x_{i}$ represents the *i*th gene position of the chromosome to be updated; $best_{i}$ is the *i*th gene position of the best chromosome in the current generation; $f_{i}$ and $f_{best}$ are the fitness values of the chromosome to be updated and the best chromosome in the current generation, respectively; $s(\alpha_{i},\beta_{i})$ indicates the rotation direction of the quantum rotation gate, which ensures that the chromosome to be updated rotates toward the direction of the best individual.

The proposed method adjusts the quantum bit amplitudes $\alpha_{i}$ and $\beta_{i}$ at the corresponding positions by comparing the fitness value of the chromosome to be updated with that of the best individual in the current generation each time. The updated probability amplitudes are denoted by $\alpha_{i}^{\prime }$ and $\beta_{i}^{\prime }$, and the entire process can be expressed as follows:

(10) $$\left[\begin{array}{c} \alpha_{i}^{\prime }\\ \beta_{i}^{\prime } \end{array}\right]=U\left(\Delta \theta \right)\left[\begin{array}{c} \alpha_{i}\\ \beta_{i} \end{array}\right].$$

Each qubit of each individual in the population satisfies the normalization condition, so the above-mentioned formula can also be rewritten as follows:

(11) $$\left[\begin{array}{c} \alpha_{i}^{\prime }\\ \beta_{i}^{\prime } \end{array}\right]=\left[\begin{array}{cc} \cos(\Delta \theta ) & -\sin(\Delta \theta )\\ \sin(\Delta \theta ) & \cos(\Delta \theta ) \end{array}\right]\left[\begin{array}{c} \cos(\theta )\\ \sin(\theta ) \end{array}\right]=\left[\begin{array}{c} \cos(\theta +\Delta \theta )\\ \sin(\theta +\Delta \theta ) \end{array}\right].$$

According to the above-mentioned expression, the quantum chromosome after rotation update still satisfies the normalization condition. Moreover, the sign and magnitude of the rotation angle Δθ have a significant impact, playing a crucial role. Namely, if the rotation angle is too small, that is, the adjustment amplitude is too small, the search range can be reduced, which may result in slow convergence of the algorithm; by contrast, if the rotation angle is too large, then the adjustment amplitude is also too large, and the search range will be expanded, which may cause the algorithm to experience premature convergence and miss the optimal solution.

Considering that the quantum chromosome length in this paper is 40, which represents $2^{40}$ possible solutions, searching for the optimal solution in such a complex search space is challenging. Therefore, this paper employs the proposed AQEA method to divide the genes in the quantum chromosome into two parts, and applies two dynamic rotation angle strategies to the probability amplitudes of qubits in the first and second halves of the genes.

For the first half of the genes, the rotation angle is calculated by

(12) $$\Delta \theta_{1}=0.04\pi \left(1-k_{1}\cdot \frac{n}{MaxGen+1}\right),$$

where *n* represents the current generation of the evolution process, MaxGen is the maximum number of evolution generations, and $k_{1}\in \left[0,1\right]$.

According to the above-mentioned formula, the rotation angle is related to the number of evolution generations. As the number of evolution generations increases, the rotation angle decreases, and the search space reduces, eventually reaching the convergence state.

For the latter half of the genes, the rotation strategy is defined as follows:

(13) $$\Delta \theta_{2}=\theta_{min}+k_{2}\cdot \frac{f_{max}-f_{i}}{f_{max}}\left(\theta_{max}-\theta_{min}\right),$$

where $\theta_{min}=0.001\pi$, $\theta_{max}=0.05\pi$; $f_{i}$ represents the fitness value of the individual to be updated; $f_{max}$ is the fitness value of the best individual in the current generation; $k_{2}$ is a natural number.

When the difference between the $f_{i}$ and $f_{max}$ values is very small and the $f_{max}$ value is large, the value of $\frac{f_{max}-f_{i}}{f_{max}}$$\left(\theta_{max}-\theta_{min}\right)$ will be much less than 0.001π, and the overall rotation angle will be less than 0.002π. This will significantly reduce the search range, making the algorithm prone to falling into a local optimum and being unable to escape from that state. However, by introducing $k_{2}$, which is a fixed value and can be dynamically adjusted during the evolutionary process, and by setting its value appropriately, it can be ensured that the rotation angle is not too small even when the $f_{i}$ value is close to the $f_{max}$ value. This can maintain an exploration ability and effectively alleviate the problem of a narrow search range caused by a small rotation angle, thus improving the overall performance of the algorithm. Based on all the aforementioned, the quantum population updating algorithm is developed, and its pseudo-code is given in Algorithm 3.

In Algorithm 3, the update rule based on the two rotation strategies is improved. Namely, when the individual to be updated is not the optimal individual, but some of its genes are the same as those of the best individual at the corresponding positions, then the probability amplitude of the corresponding individual in the previous generation can be directly continued, and there is no need to perform rotation updates. Only the gene positions with different states from those of the best individual need to be used for optimization. This not only simplifies the judgment logic but also reduces the implementation complexity of the algorithm. In addition, since the quantum rotation strategy targets only half of the genes at each iteration, the other half of the genes directly copy the state of the previous generation.
**Algorithm 3** Quantum population updating.**Input:** An evaluated quantum population *Q*, a size of quantum population *N*, the length of a quantum chromosome *L*, the probability of mutation $P_{mutation}$, a catastrophic factor $C_{factor}$, the population fitness value *F*, the maximum generation *T***Output:** updated quantum population $Q^{\prime }$ 1:Rank population individuals $Q\to Q_{rank}$ according to their fitness values 2:Record the fitness value of the best individual $F_{best}$ 3:**if** count of $F_{best}<C_{factor}*T$ **then** 4:      **for** i=1→2/L **do** 5:            **if** $F_{i}<F_{best}$ **then** 6:                 **if** $Q_{i}\neq Q_{best}$ **then** 7:                      Update the population using the first quantum rotation strategy 8:                 **end if** 9:            **else**10:                 Initialize the probability *P* randomly in the range of (0,1) and mutate11:            **end if**12:      **end for**13:      Obtain quantum subpopulation $Q_{1}$14:      **for** i=2/L→L **do**15:            **if** $F_{i}<F_{best}$ **then**16:                 **if** $Q_{i}\neq Q_{best}$ **then**17:                      Update the population using the second quantum rotation strategy18:                 **end if**19:            **else**20:                 Initialize the probability *P* randomly in the range of (0,1) and mutate21:            **end if**22:      **end for**23:      Obtain quantum subpopulation $Q_{2}$24:      Merge quantum subpopulation $Q_{1}$ and $Q_{2}$ to update $Q^{\prime }$25:**else**26:      Retain the best individual $F_{best}$ and reinitialize the other individuals27:**end if**28:Return updated quantum population $Q^{\prime }$

## 4. Experimental Verification

This section mainly focuses on information on the experiments conducted in this study, including the introduction of the experimental datasets, experimental environment, experiment settings, and experimental results and their comparison analysis.

### 4.1. Datasets

The datasets used in the experiments in this study included the MNIST, Fashion-MNIST, and CIFAR-10 datasets, ensuring a diverse range of data for comprehensive evaluation. The three datasets are the most commonly used image classification datasets in the fields of machine learning and deep learning; they cover the task requirements from basic to advanced levels and provide important benchmarks for the development and evaluation of image classification models. To verify the classification results of the quantum circuits based on the AQEA search and compare them with those of the EQNAS [50], three binary classification tasks were selected and performed. The three classification tasks included: the digits {3, 6} in the MNIST dataset; the “T-shirt” and “dress” in the Fashion-MNIST dataset, with labels {0, 3}; the “cat” and “dog” in the CIFAR-10 dataset, with labels {3, 5}.

### 4.2. Experimental Setup

The QNN search based on the proposed AQEA was implemented in the PyTorch (2.4.1) machine-learning framework. The construction and training of quantum circuits were performed on the quantum simulator PennyLane (0.38.0). The experimental equipment included the NVIDIA GeForce GTX4090 GPU. To ensure the fairness and impartiality of the experiment, the population size in both the AQEA and the QEA was set to four, the length of a quantum chromosome was set to 40, the number of evolutions was set to 10, and the initial mutation probability of individuals in the population was set to 0.5. In addition, in the AQEA, the quantum population catastrophe factor was set to 0.4, indicating that when the highest fitness individual remained unchanged for more than 10 × 0.4 = 4, a catastrophe was initiated. The individual with the highest fitness value in the current generation and its fitness value were retained, whereas the other individuals were reinitialized, and the mutation probability of the retained individual increased. The minimum and maximum angles in the dynamic rotation strategy were 0.0001π and 0.05π, respectively. Due to the limitation in quantum bit resources, the number of quantum bits used in the quantum circuits was set to 10. The same number of quantum bits was also used in the AQEA. The dataset was scaled to a size of 2 × 5. It should be noted that since the CIFAR-10 dataset included color images, it was necessary to convert the images into gray-scale images first and then send them to the variational layer mentioned in the previous chapter. In the model training process, the Adam optimization algorithm was used, with an initial learning rate of 0.003, a weight decay of 0.00001, 20 training epochs, and a batch size of 32.

### 4.3. Experimental Results and Analysis

#### 4.3.1. Comparison with EQNAS and QNN

To verify the optimization effects of the AQEA and QEA on the QNN structure [54], this study compared the performance of the two optimization algorithms and the original QNN in classification tasks. The parameter layer of the QNN contained a layer of XX gates and a layer of ZZ gates; the connection method defined that the zeroth to ninth qubits were sequentially connected to the 10th auxiliary qubit, totaling 20 quantum gates and 20 parameters.

The results of the classification performance of the QNN and the quantum circuits optimized by the QEA and AQEA on the three datasets are presented in Table 3. As shown in Table 3, the number of quantum gates used in the three structures was larger by 23 than the number of parameters, which was due to the addition of the rotation gates used in the previous quantum encoding process and the three quantum gates of the auxiliary qubits. In addition, the results indicated that the optimized QNN structure could not only reduce the number of quantum gates but also improve the accuracy. Particularly, the structure optimized by the AQEA’s accuracy was 7.21% higher than that of the QNN on the MNIST dataset and 4.57% higher than that optimized by the QEA. The EQNAS had the same number of parameters as the QNN, whereas the AQEA-QNN had 20% fewer parameters compared with the EQNAS. On the Fashion-MNIST and CIFAR-10 datasets, the model size and accuracy of the EQNAS were almost the same as those of the QNN. By contrast, the AQEA-QNN has 20% fewer parameters than the QNN on complex datasets containing more feature information and 11.11% and 20% fewer parameters than the QEA-QNN. The proposed AQEA-QNN also increased the accuracy by 3.95% and 2.35% compared with the QNN and by 3% and 2% compared with the EQNAS. This could be attributed to the two adaptive rotation strategies of the AQEA, which could explore a broader search space and find more efficient and stable structures than the QEA.

**Figure 5:**
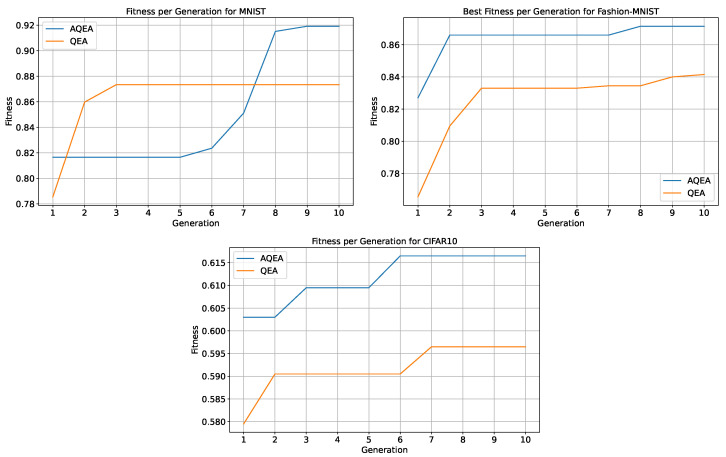
The fitness curves of the QEA and AQEA on the three datasets. It can be seen that the blue line is mostly above the orange line, indicating that AQEA searches for populations with higher quality within the same number of evolutionary iterations.

**Figure 6:**
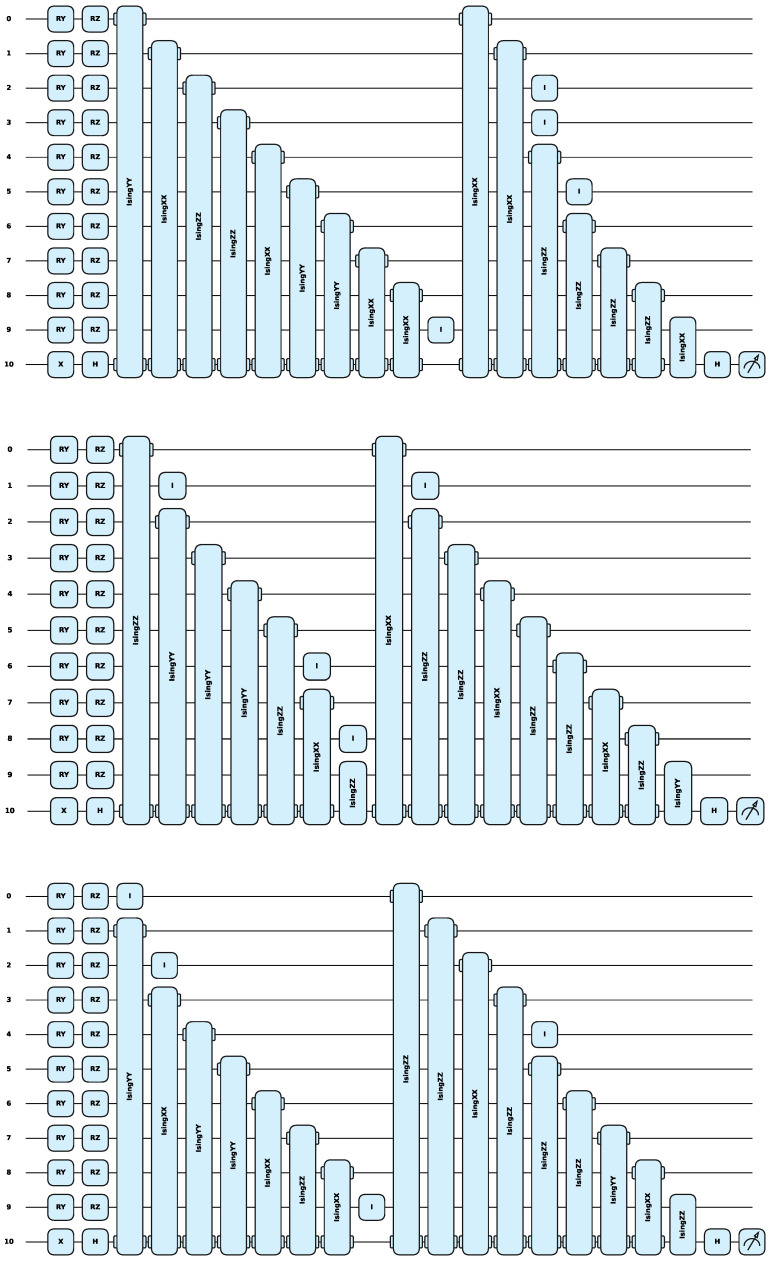
From top to bottom are the optimal circuit architectures on the MNIST, Fashion-MNIST, and CIFAR-10 datasets. It can be seen that the parameters of the searched circuit architecture are fewer, with only 16 parameters.

For the two evolutionary algorithms, the QEA and AQEA, this study evaluated the quantum chromosomes generated by the two algorithms and the individuals decoded by them and plotted the fitness curves, as shown in Figure 5. The results indicated that the quantum population quality obtained by the AQEA was significantly higher than that obtained by the QEA, and the fitness of the AQEA on the three datasets was above that of the QEA. On the latter two datasets, the fitness of individuals with the highest fitness in each generation obtained by the AQEA gradually increased with algebra, and almost every generation was higher compared with the QEA. Because of the uncontrollability of variation, there was not always an individual who was better than the parent in the next generation, so the highest fitness of successive generations was the same individual. However, a quantum cataclysm operation was added to the proposed AQEA, which made the proposed algorithm more likely to escape the current solution in the case of falling into a local optimum compared with regular QEA. The evaluation results on the MNIST dataset demonstrated that the population fitness of the AQEA-QNN changed multiple times, and finally reached an accuracy of 91.92% on the test dataset. However, the QEA underwent only two changes, with the highest fitness reaching only 87.35%. This revealed that the AQEA had a faster search speed than the QEA when the population size and evolution times were the same; it could search for more suitable solutions to the current problem in a massive search space and had a faster convergence speed. Circuits are shown in Figure 6.

#### 4.3.2. Comparison with Other Quantum Circuits

In addition to the above-presented comparison analysis with the QNN and EQNAS, this study also conducted comparison experiments with the hybrid networks of the same order of magnitude. In these experiments, the process of quantum coding remained the same as in the previous experiments; these experiments mainly tested the classification ability of different artificially designed quantum circuits and added a fully connected layer to the end of the circuit for classification.

**Figure 7:**
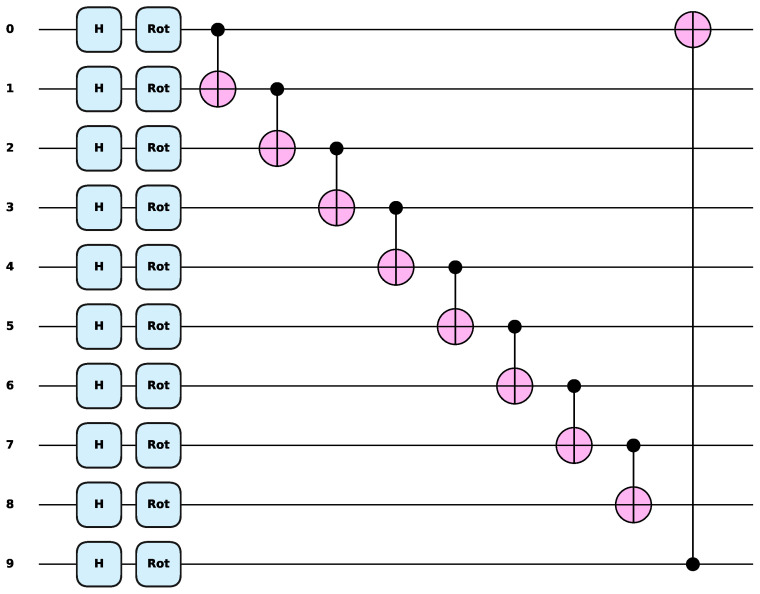
The PQC employed in the VQDNN. The Rot gate consists of three quantum gates, $R_{z}$, $R_{y}$, and $R_{z}$ gate, and one Rot gate contains 3 parameters. The CNOT gate is responsible for creating entanglement among all qubits.

In these experiments, parametric quantum circuits in the VQDNN proposed in [55] were first used. The PQC employed in the VQDNN is displayed in Figure 7, where it can be seen that it includes the Hadamard gate, Rot gate, and CNOT gate. Only the Rot gate had three parameters for a total of 10 qubits, which corresponded to 30 training parameters. This structure was then used for training and testing on the three datasets, namely, the MNIST, Fashion-MNIST, and CIFAR-10 datasets.

**Figure 8:**
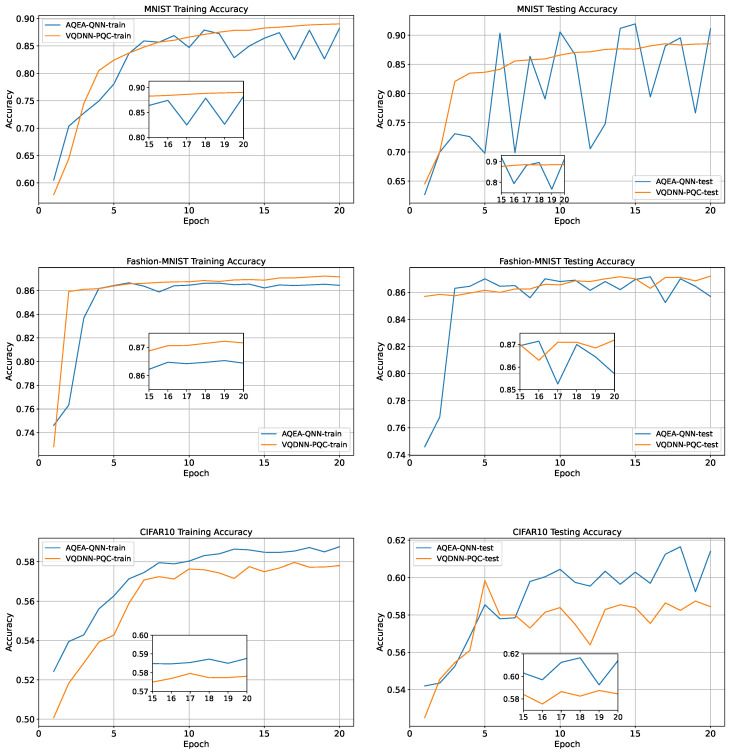
The training and testing curves of the AQEA-QNN and VQDNN-PQC. It can be observed that even with more than 20 fewer parameters, the accuracy remains higher.

The training and testing curves of the two models are depicted in Figure 8, where it can be observed that on the MNIST and CIFAR-10 datasets, the accuracy of the AQEA-QNN on the test dataset was higher than that of the VQDNN-PQC by 3.4% and 2.9%, respectively. In addition, the VQDNN-PQC had 3×10 + 11 = 41 parameters, where the 11 parameters denoted the 10 weights and one bias of the subsequent fully connected network. However, the AQEA-QNN had 16 parameters, which denoted less than half of the VQDNN-PQC’s parameter number. However, on the Fashion-MNIST dataset, the number of parameters of VQDNN-PQC is 25 more than that of AQEA-QNN, but its accuracy is only 0.05% higher. The results are presented in Table 4.

**Figure 9:**
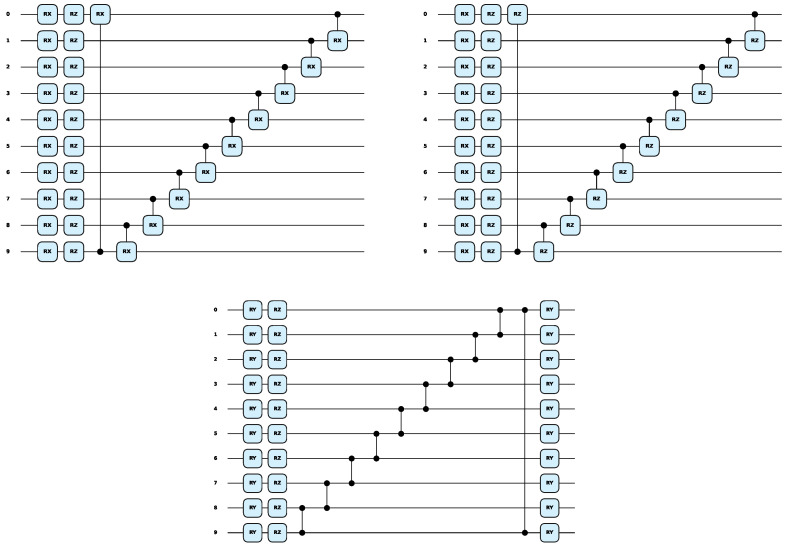
The three typical template circuits presented in [56]. They are ansatz1, ansatz2, and ansatz3 from left to right. These circuit templates are from or inspired by past studies.

**Figure 10:**
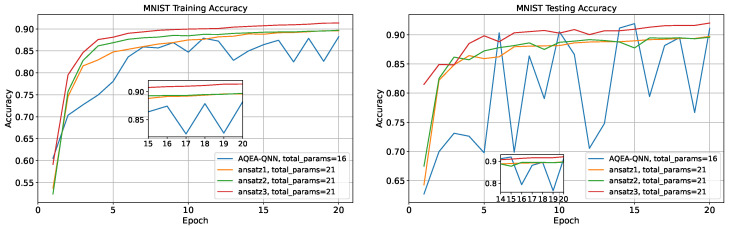

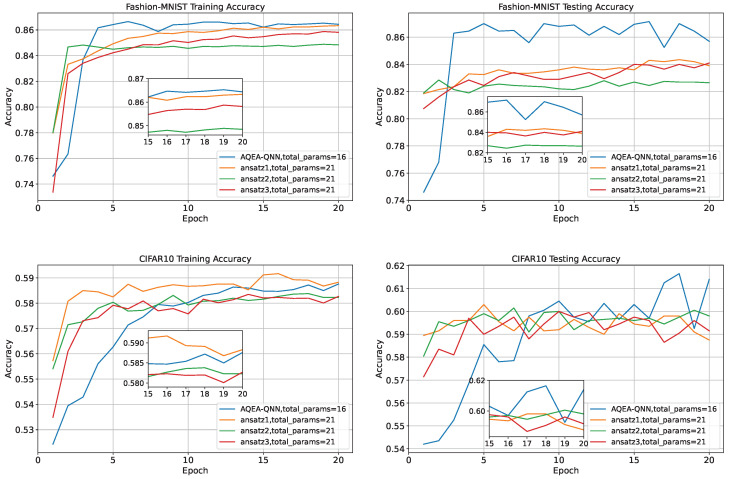
The training and testing curves of the AQEA-QNN, ansatz1, ansatz2, and ansatz3. It can be seen that the performance on the last two datasets is significantly better than that of general ansatzes, with the highest improvement reaching 3.05%.

Next, these template circuits were used for comparison analysis, as shown in Figure 9, which were derived from or inspired by previous research in the field. The $R_{x}$ and $R_{z}$ gates before ansatz1 and ansatz2 were repeated with the previous coding process; the original line was used here, and the total parameter number was 10 + 11 = 21. The performance of the ansatzes on the three datasets is depicted in Figure 10. The results indicated that, except for ansatz3, which had a slightly higher effect than the AQEA-QNN on the test dataset of the MNIST dataset, the other template circuits had a lower effect than the AQEA-QNN on all datasets but with more parameters. Thus, the efficient circuit structure was more dominant than the parameters. In addition, the learning curve of the AQEA-QNN was smoother than that of the three ansatzes, which indicated that the circuit architecture of the AQEA-QNN was better than that of the template circuit for capturing advanced and complex features. This also demonstrated the importance of selecting an appropriate circuit architecture. Moreover, it can be seen that the accuracy of ansatz3 on the MNIST dataset was higher than that of ansatz1 but lower on the Fashion-MNIST and CIFAR-10 datasets. The specific results are presented in Table 5. The results showed that it was difficult for the same circuit model to achieve good results in different classification tasks and validated the necessity of the AQEA in the search for a quantum circuit architecture.

### 4.4. Adjustment of AQEA Parameters

In AQEA, the fine-tuning process of parameters is critical as these parameters directly govern the algorithm’s exploration efficiency, generalization capability, and overall performance. To identify the optimal configuration and validate its effectiveness, this study conducted extensive experiments to systematically evaluate the impact of different parameter combinations. By meticulously adjusting the population size *N*, generation count *T*, and rotation angle parameters $k_{1}$ and $k_{2}$, this paper identified that the configuration N=4, T=10 achieves a balance between computational efficiency and maximized expressive power across diverse datasets. Experimental results from Table 6 demonstrate that this parameter configuration not only improves the model’s fitness value (i.e., optimization objective performance) but also ensures algorithmic stability and robustness across heterogeneous problem scenarios on benchmark datasets including MNIST, Fashion-MNIST, and CIFAR-10. Notably, the smaller *N* significantly reduces per-iteration time costs, while the appropriate *T* guarantees sufficient exploration of the solution space. Furthermore, the carefully selected rotation angle parameters enhance the algorithm’s ability to escape local optima, facilitating the discovery of global optimal solutions.

### 4.5. Noise Analysis in Quantum Systems

Quantum noise constitutes a fundamental challenge in quantum computing systems, as it not only imposes limitations on the precise manipulation of quantum states but also directly impacts the reliability and computational capabilities of quantum circuits. With the advancement of quantum computing technologies, quantum noise has emerged as a critical metric for evaluating the quality of quantum circuit architectures. Quantum circuit fidelity, serving as the key parameter for assessing quantum computing system performance, directly reflects the accuracy of quantum operations. Furthermore, the selection of circuit architectures profoundly influences both the pattern and magnitude of noise accumulation.

Quantum noise primarily manifests through four fundamental forms: bit flip noise, phase flip noise, amplitude damping noise, and depolarizing noise. Bit flip (BF) noise corresponds to the Pauli-X operation, which interconverts the quantum states |0〉 and |1〉, analogous to classical bit flip errors. Phase flip (PF) noise is associated with the Pauli-Z operation, preserving the amplitude of quantum states while altering their relative phase coherence—for instance, transforming $|+\rangle =\frac{1}{\sqrt{2}}(|0\rangle +\left|1\rangle \right)$ into $|-\rangle =\frac{1}{\sqrt{2}}(|0\rangle -\left|1\rangle \right)$, thereby disrupting quantum coherence. Amplitude damping (AD) noise describes the energy dissipation process in quantum systems, typically represented by the non-unitary evolution from the |1〉 state to the |0〉 state. Its Kraus operators are expressed as:

(14) $$E_{0}=\left(\begin{array}{cc} 1 & 0\\ 0 & \sqrt{1-\gamma } \end{array}\right),\;E_{1}=\left(\begin{array}{cc} 0 & \sqrt{\gamma }\\ 0 & 0 \end{array}\right),$$

where γ quantifies the energy loss rate. Depolarizing noise (DN), the most universal noise model, stochastically maps quantum states to any operator in the Pauli set {I,X,Y,Z} with probability *p*, resulting in reduced quantum state purity. The mathematical formulation is:

(15) $$\rho \to \left(1-p\right)\rho +\frac{p}{3}\left(X\rho X+Y\rho Y+Z\rho Z\right),$$

where ρ denotes the density matrix. Collectively, these four noise archetypes constitute the dominant error sources in quantum computing systems.

Quantum fidelity serves as a critical metric for evaluating quantum state quality, quantifying the similarity between two quantum states. For pure states |ψ〉 and |ϕ〉, the fidelity is defined as:

(16) $$F(|\psi \rangle ,|\phi \rangle )={\left|\langle \psi |\phi \rangle \right|}^{2},$$

while for mixed states ρ and σ, it adopts the more complex form:

(17) $$F\left(\rho ,\sigma \right)={\left[\mathrm{Tr}\left(\sqrt{\sqrt{\rho }\;\sigma \sqrt{\rho }}\right)\right]}^{2}.$$

Quantum noise and quantum fidelity share an intrinsic connection: noise acting through quantum channels induces deviations in quantum state evolution from ideal trajectories, thereby reducing the fidelity between actual and target states, which establishes fidelity as the central metric for quantifying noise-induced degradation in quantum systems.

According to the data in Table 7, the performance of the optimal circuit architecture under different noise types and intensities is significantly superior to that of the traditional ansatz structures, with its advantages mainly reflected in the noise suppression capability of the structural design. Taking PF as an example, when the noise probability p=0.5, the fidelity of the optimized structure for MNIST still remains at 0.8314, while those of ansatz1 and ansatz2 decrease to 0.4853 and 0.4335, respectively, with a reduction of 33% and 38% approximately, indicating that the optimized structure effectively suppresses the propagation path of phase noise through gate count optimization and connectivity adjustment. In the case of BF, the fidelity of ansatz1 almost drops to zero at p=0.5, while the optimized structure only decreases to 0.8398, illustrating that it reduces the cumulative effect of bit flips by minimizing the use of improper gates with high error rates. Additionally, AD and DN noises, the performance decay amplitude of the optimized structure is also significantly smaller than that of the traditional structures. These data demonstrate that the optimized structure, through AQEA optimization, effectively reduces the impact of noise on circuit performance.

## 5. Conclusions and Future Work

This study proposes an innovative adaptive quantum evolution algorithm named AQEA to optimize the quantum circuit architecture, aiming to obtain a simple structure and strong expression of the circuit structure. The proposed algorithm includes three stages. The first stage contains the initialization and decoding processes of quantum chromosomes, which convert quantum chromosomes into classical chromosomes. The second stage creates quantum circuits based on the observed chromosome structure, which are then evaluated on a test dataset using a quantum circuit model. The last stage is the regeneration stage of the quantum population. This study introduces two dynamic adaptive rotation strategies, of which one acts on the first half of the quantum chromosome genes and the second half of the genes. In addition, one of the two dynamic renewal strategies is related to the number of evolution, which decreases with the increase in the evolutionary algebra, and the other strategy is related to the highest fitness value of each generation, and each non-optimal individual will evolve in the direction of the optimal individual. Moreover, quantum catastrophes are introduced to prevent local optimality. Namely, when the optimal individual does not change for several generations, catastrophe is initiated, and then the structure and fitness of the optimal individual are retained, whereas the other individuals are reinitialized; these steps are repeated until the iteration limit is reached. The experimental results show that compared with the QEA-QNN, the original QNN, and some other proposed template circuits, the number of quantum gates of the proposed algorithm is reduced by 17.39%, the number of parameters is decreased by 20%, and the accuracy is improved by 7.21%. In noise experiments, the circuit optimized by AQEA outperforms traditional templates significantly. Under various noises, it maintains high fidelity, demonstrating its excellent noise-resistant ability and ensuring the reliability of quantum computing in noisy conditions.

Although the proposed algorithm has achieved good results, more complex adaptive mechanisms can still be explored. Strategies such as machine learning can be integrated into the core rotation strategy optimization to enhance performance, and a multi-objective framework can be adopted to consider factors like execution time, ensuring algorithm stability. Traditional PQCs enhance expressiveness via multi-layer gate stacking: while increasing layers better approximates the objective function, it introduces challenges like noise. Integrating efficient quantum compilation techniques with advanced processors enhances experimental reliability and facilitates the algorithm’s application. Additionally, research into using genetic programming to decompile low-level circuits for architectural interpretability enhancement offers new insights for current work. When searching for quantum circuit architectures, this technique can be used to analyze circuits, clarify gate functions in machine learning, assist in algorithmic search path analysis, and integrate interpretability into multi-objective optimization, thereby enabling quantum circuits to achieve better performance in complex tasks by coordinating with metrics like execution efficiency.

## Figures and Tables

**Table 1:** The decoding methods of quantum chromosomes.

Two-Qubits State	Quantum Gate
|00〉	XX
|01〉	YY
|10〉	ZZ
|11〉	*I* (Identity)

**Table 2:** The lookup table of the rotation angle.

				$s(\alpha_{i},\beta_{i})$			
$x_{i}$	${\mathrm{best}}_{i}$	$f_{i}>f_{\mathrm{best}}$	Δθ	$\alpha_{i}\beta_{i}>0$	$\alpha_{i}\beta_{i}<0$	$\alpha_{i}=0$	$\beta_{i}=0$
0	0	False	0	0	0	0	0
0	0	True	0	0	0	0	0
0	1	False	0	0	0	0	0
0	1	True	0.01π	−1	+1	±1	0
1	0	False	0.01π	−1	+1	±1	0
1	0	True	0.01π	+1	−1	0	±1
1	1	False	0.01π	+1	−1	0	±1
1	1	True	0.01π	+1	−1	0	±1

**Table 3:** Comparison with EQNAS and QNN.

Model	MNIST	Fashion-MNIST	CIFAR-10	Q-Gates	Params
QNN [54]	84.71%	83.20%	59.30%	43	20
EQNAS [50]	87.35%	84.15%	59.65%	43,41,43	20,18,20
AQEA-QNN	91.92%	87.15%	61.65%	39,39,39	16,16,16

**Table 4:** Comparison with the VQDNN using the circuit displayed in Figure 7.

Model	Method	MNIST	Fashion-MNIST	CIFAR-10	Params	Q-Gates
VQDNN [55]	Manual	88.52%	87.20%	58.75%	30 ^1^ + 11 ^2^	50
AQEA-QNN	AQEA	91.92%	87.15%	61.65%	16 ^1^,16 ^1^,16 ^1^	39,39,39

^1^ Quantum gate parameters. ^2^ FC layer parameters.

**Table 5:** Comparison with three different template circuits presented in [56].

Model	Method	MNIST	Fashion-MNIST	CIFAR-10	Params	Q-Gates
ansatz1	Manual	89.74%	84.35%	60.30%	10 ^1^ + 11 ^2^	30
ansatz2	Manual	89.58%	82.85%	60.15%	10 + 11	30
ansatz3	Manual	92.02%	84.10%	60.00%	10 + 11	40
AQEA-QNN	AQEA	91.92%	87.15%	61.65%	16 ^1^,16 ^1^,16 ^1^	39,39,39

^1^ Quantum gate parameters. ^2^ FC layer parameters.

**Table 6:** Impact of changes in significant parameters in AQEA.

Dataset	Population	Rotation Parameters	Fitness
MNIST	$N^{\;1}=4$, $T^{\;2}=10$	${k_{1}}^{\;3}=0.1$, ${k_{2}}^{\;4}=1$	0.8328
		$k_{1}=0.1$, $k_{2}=2$	0.9096
		$k_{1}=0.1$, $k_{2}=3$	0.8984
		$k_{1}=0.2$, $k_{2}=2$	0.8902
		$k_{1}=0.05$, $k_{2}=2$	0.9192
	N=6, T=15	$k_{1}=0.1$, $k_{2}=2$	0.8720
	N=8, T=20	$k_{1}=0.1$, $k_{2}=2$	0.8943
Fashion-MNIST	N=4, T=10	$k_{1}=0.1$, $k_{2}=1$	0.8655
		$k_{1}=0.1$, $k_{2}=2$	0.8660
		$k_{1}=0.1$, $k_{2}=3$	0.8715
		$k_{1}=0.2$, $k_{2}=2$	0.8660
		$k_{1}=0.05$, $k_{2}=2$	0.8655
	N=6, T=15	$k_{1}=0.1$, $k_{2}=2$	0.8705
	N=8, T=20	$k_{1}=0.1$, $k_{2}=2$	0.8710
CIFAR-10	N=4, T=10	$k_{1}=0.1$, $k_{2}=1$	0.6125
		$k_{1}=0.1$, $k_{2}=2$	0.6165
		$k_{1}=0.1$, $k_{2}=3$	0.6080
		$k_{1}=0.2$, $k_{2}=2$	0.6145
		$k_{1}=0.05$, $k_{2}=2$	0.6145
	N=6, T=15	$k_{1}=0.1$, $k_{2}=2$	0.6125
	N=8, T=20	$k_{1}=0.1$, $k_{2}=2$	0.6100

^1^ Size of quantum population. ^2^ The maximum generation. ^3^ Coefficient of (12). ^4^ Coefficient of (13).

**Table 7:** Fidelity of AQEA-QNN and template circuits under noisy and noise-free environments.

Model	Noise-Free Fidelity	Noise Type	*p* ^1^	Fidelity
Optimal circuit architecture on MNIST	0.8516	PF	0.1	0.8237
			0.5	0.8314
		BF	0.1	0.8334
			0.5	0.8398
		AD	0.1	0.8298
			0.5	0.8396
		DN	0.1	0.8325
			0.5	0.8289
Optimal circuit architecture on Fashion-MNIST	0.8346	PF	0.1	0.8310
			0.5	0.8312
		BF	0.1	0.8328
			0.5	0.8308
		AD	0.1	0.8319
			0.5	0.8264
		DN	0.1	0.7740
			0.5	0.8291
Optimal circuit architecture on CIFAR-10	0.8265	PF	0.1	0.8207
			0.5	0.8310
		BF	0.1	0.8222
			0.5	0.8337
		AD	0.1	0.8201
			0.5	0.8366
		DN	0.1	0.8204
			0.5	0.8283
ansatz1	0.7238	PF	0.1	0.5627
			0.5	0.4853
		BF	0.1	0.1315
			0.5	0.0048
		AD	0.1	0.4296
			0.5	0.0509
		DN	0.1	0.2063
			0.5	0.0080
ansatz2	0.6989	PF	0.1	0.5103
			0.5	0.4335
		BF	0.1	0.1279
			0.5	0.0049
		AD	0.1	0.4087
			0.5	0.0493
		DN	0.1	0.1931
			0.5	0.0078

^1^ Noise intensity.

## Data Availability

The detailed code for this study can be found at https://github.com/918939601/AQEA-QAS (accessed on 1 July 2025).
